# Supplementary material for: Effects of Selenium Yeast on Egg Quality, Plasma Antioxidants, Selenium Deposition and Eggshell Formation in Aged Laying Hens
Source: Animals (Basel). 2023 Mar 1;13(5):902. doi: 10.3390/ani13050902 (PMC10000209; doi:10.3390/ani13050902)
Supplement: Supplementary file 1 [file animals-13-00902-s001.zip › Supplementary Table S1.pdf]

Table S1. Primers used for qRT-PCR.

Gene	Sequence (5′-3′)	Product length, bp	Annealing temperature, ℃
OVAL	F: AAGCAGGCAGAGAGGTGGTAGG<br>R: ACGGCGTTGGTTGCGATGTG	121	62
SLC6A17	F: TCAAGGTGCGGAAGGAGGTGTT<br>R: ACGGCGATGTTCTCCAGGATGA	163	64
CEMIP	F: TCTTCAGCGACCGTCCACTCAA<br>R: CTGCCGATCCGTAGCTCTCCAT	131	58
β-actin	F: GAGAAATTGTGCGTGACATCA<br>R: CCTGAACCTCTCATTGCCA	152	56

Abbreviations: *OVAL* = ovalbumin; *SLC6A17* = solute carrier family 6 member 17; *CEMIP* = cell migration inducing hyaluronidase 1.
