# Supplementary figures and images for: Effects of Selenium Yeast on Egg Quality, Plasma Antioxidants, Selenium Deposition and Eggshell Formation in Aged Laying Hens
Source: Animals (Basel). 2023 Mar 1;13(5):902. doi: 10.3390/ani13050902 (PMC10000209; doi:10.3390/ani13050902)

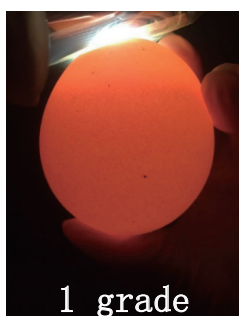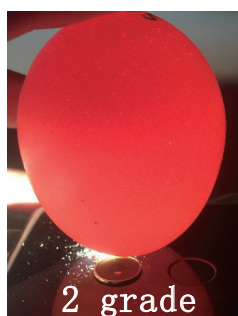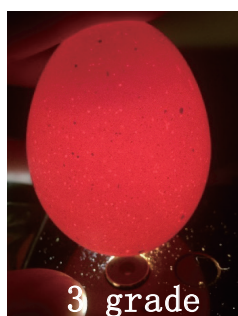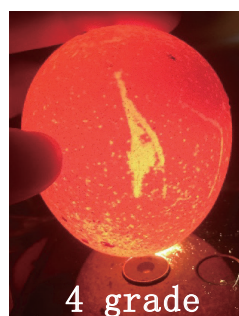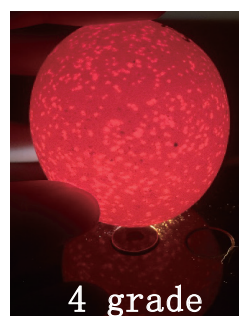

Supplement: Supplementary file 1 [file animals-13-00902-s001.zip › Figure S1.pdf]
